# Memory representations in a cross-modal matching task: evidence for a verbal component

**DOI:** 10.3389/fpsyg.2023.1253085

**Published:** 2023-11-23

**Authors:** Katherine Marie Estabrooks, Muhammad Tayyab Sohail, Young In Song, Geneviève Desmarais

**Affiliations:** Department of Psychology, Mount Allison University, Sackville, NB, Canada

**Keywords:** multisensory integration, cognitive styles, memory representations, visual perception, haptic perception, object matching

## Abstract

In everyday tasks, one often uses touch to find what has been seen. Recent research has identified that when individuals view or touch an object, they may create a verbal memory representation; however, this research involved object naming, which may have prompted the use of verbal strategies. Research has also identified variability in memory representations for objects, which may indicate individual differences. To investigate memory representations and their associations with individual differences in cognitive styles, we measured the cognitive styles of 127 participants and had them complete a non-verbal matching task without distractors, or with verbal or visual distractors. In the task, they viewed an object and then touched an object – or vice versa - and indicated whether the objects were the same or different. On trials where different objects were presented, participants responded consistently more slowly and made more matching errors for similar objects compared to distinct objects. Importantly, higher scores on the verbalizer cognitive style predicted faster reaction times on the matching task across all trial types and distraction conditions. Overall, this indicates that cross-modal object processing in short-term memory may be facilitated by a verbal code.

## Introduction

Individuals sometimes need to use touch to find a previously seen object, for example feeling through a full school bag for a pen. To perform these tasks, information taken in visually is used to later recognize something haptically; this is cross-modal processing. Visual and haptic recognition rely on similar object properties. Cooke et al. (2007) found that regardless of whether participants categorized novel objects visually or haptically, they relied primarily on shape and texture information. This was also observed for haptic identification (Klatzky et al., 1985), and for natural objects (Gaißert et al., 2011; Gaißert and Wallraven, 2012). It therefore seems that categorizing or recognizing objects by sight or touch relies on object shape. Further, in these studies objects were categorized into similar clusters independently of modality - visual and haptic processes for categorization are therefore similar. Consequently, it is not surprising that information learned in one modality is available to the other modality.

Cross-modal processing applies to object processing. Desmarais et al. (2017b) and Desmarais and Penrose (2021) demonstrated this when participants learned to recognize novel objects by sight and were later able to identify the objects by touch, and vice versa. Cross-modal processing has also been demonstrated for 2-D and 3-D objects (Easton et al., 1997a), natural objects (Norman et al., 2004), scenes (Newell et al., 2005) and letters (Easton et al., 1997b). Together, these studies show that information encoded visually or haptically can be accessed by the other modality, suggesting shared memory representations (Easton et al., 1997b; Norman et al., 2004; Desmarais et al., 2017b; Desmarais and Penrose, 2021). However, these studies do not provide evidence as to what kinds of memory representations are created.

Since object processing seems to rely on shape information (Klatzky et al., 1987; Norman et al., 2004; Cooke et al., 2007; Desmarais et al., 2017b; Desmarais and Penrose, 2021), we can expect object memory representations to include a visual component. Indeed, visual representations seem to be recruited for novel objects. Lacey and Campbell (2006) demonstrated that participants’ cross-modal recognition performance was less accurate when they were presented with visual distractors, suggesting that visual memory representations are used in cross-modal object processing. Furthermore, Desmarais et al. (2017b) and Desmarais and Penrose (2021) observed that errors in object identification were driven by the visual similarity between objects. This suggests that the visual and haptic modality share visual representations.

There is also evidence for the use of verbal representations in object processing. Lacey and Campbell (2006) demonstrated that verbal distractors decreased cross-modal recognition accuracy; suggesting that verbal interference hindered the creation of object representations. This is confirmed by Desmarais and Penrose (2021), who found that when participants learned to recognize objects by sight or touch, only verbal distractors interfered with object identification. However, the use of a naming task in Desmarais and Penrose (2021) may have biased participants toward using a verbal code. Additionally, Desmarais et al. (2017a) had participants learn to associate actions and verbal labels with novel objects, and later identify the objects and perform the action associated with each object. The authors showed that the presence of verbal labels impacts object use, suggesting that verbal representations may be important for object processing. Together, these studies suggest the importance of verbal representations in long-term memory for cross-modal object processing.

Short-term memory and working memory also seem to rely on verbal information. Postle et al. (2005) investigated participant’s performance on an object n-back task completed without distraction, with motion or verbal distractors. Participants made more recognition errors when verbal distractors were present, suggesting that short-term memory may recruit verbal representations. Similarly, Santana and Galera (2014) asked participants whether two arrangements of four letters were the same or different. Changes were made to either the verbal dimension, the visual dimension or the spatial dimension, and participants were instructed to pay attention to one dimension and ignore the others. The results demonstrated that participants often could not ignore a change in the verbal dimension, even when instructed to ignore it. The verbal dimension was bound to the information participants intended to encode, which is evidence that working memory may recruit verbal representations. However, the findings regarding representations recruited in visuo-haptic object processing are variable, and at least one study (Lacey and Campbell, 2006) has demonstrated that participants recruited both verbal and visual representations. Therefore, some of this variability may be due to individual differences such as cognitive styles.

Cognitive styles refer to ways in which individuals take in and process information (Blazhenkova and Kozhevnikov, 2009). While individuals use multiple dimensions to process information, the cognitive style describes the dimension most consistently used to accomplish these tasks. To process information, object-visualizers tend to generate detailed mental images of individuals or objects, while spatial-visualizers tend to generate mental images that are more schematic and representative of spatial orientation or the relationship between an object and its location. On the other hand, verbalizers tend to use verbal descriptions or strategies to process information (Blazhenkova and Kozhevnikov, 2009). Cognitive styles have been shown to be associated with learning modality preference (Pazzaglia and Moè, 2013; Höffler et al., 2017) and mental abilities and task performance (Occelli et al., 2014; Pérez-Fabello et al., 2018). Most importantly, cognitive styles have been associated with strategies used during encoding and recall (Meneghetti et al., 2014), and might therefore explain some of the variability observed in object representations.

We therefore investigated whether object representations in memory were based on a visual or a verbal code, and whether this would be predicted by cognitive styles. Recall that Desmarais and Penrose (2021) used a naming task to demonstrate evidence of verbal representations, which may have biased participants toward using a verbal strategy. We therefore used the same stimuli in a matching task that did not require naming. Participants first completed the Object-Spatial Imagery and Verbal Questionnaire (OSIVQ, Blazhenkova and Kozhevnikov, 2009), followed by an object matching task. In the matching task, participants completed trials where they saw an object and then touched an object and indicated whether the two objects were the same or different, as well as trials where the order of the modalities was reversed. Importantly, one third of participants completed the task as is (control condition), while the others were presented with letter distractors (verbal distractor condition) or symbol distractors (visual distractor condition). Based on Desmarais and Penrose (2021), we hypothesized that participants in both distractor conditions would respond more slowly and make more errors than participants in the control condition. Further, we hypothesized that participants’ cognitive styles scores would predict performance. We expected that for participants in the verbal distraction condition, higher scores on the verbalizer subscale would predict slower response times and more errors, and that for participants in the visual distraction condition, higher scores on either visualizer subscales would predict slower response times and more errors.

## Methods

### Participants

One hundred and twenty-seven undergraduate participants were recruited from Mount Allison University (96 females, 27 males, *M_age_* = 19.87 years). Prior to beginning the study, we estimated the sample size using G-Power, a statistical power analysis program (see Faul et al., 2007, 2009), using the effect size η^2^ = 0.125. (Desmarais et al., 2017b) and α = 0.05. The estimated sample size was 111, which was rounded upwards to 120 (40 participants in each of the three experimental conditions). As compensation, students were given either 2.5 course credit or $24.00.

### Materials

### Object-spatial imagery and verbal questionnaire (OSIVQ)

Cognitive styles were measured using the Object-Spatial Imagery and Verbal Questionnaire (OSIVQ), a 45-item scale developed by Blazhenkova and Kozhevnikov (2009). The questionnaire required participants to rate items according to how much each statement describes them on a 5-point scale of 1 (totally disagree) to 5 (absolutely agree). Fifteen items measure each of the object-visualizer style (e.g., “I have excellent abilities in technical graphics”); the spatial-visualizer style (e.g., “I can easily sketch a blueprint for a building I am familiar with”) and the verbalizer style (e.g., “I have better than average fluency in using words”). Participants’ scores were obtained by calculating their average rating for object-visualizer, spatial-visualizer, and verbalizer scales. Internal reliability for the OSIVQ was reported by Blazhenkova and Kozhevnikov (2009) to be α = 0.74 (verbal), α = 0.83 (object), and α = 0.83 (spatial).

### Novel objects

**Figure 1:**
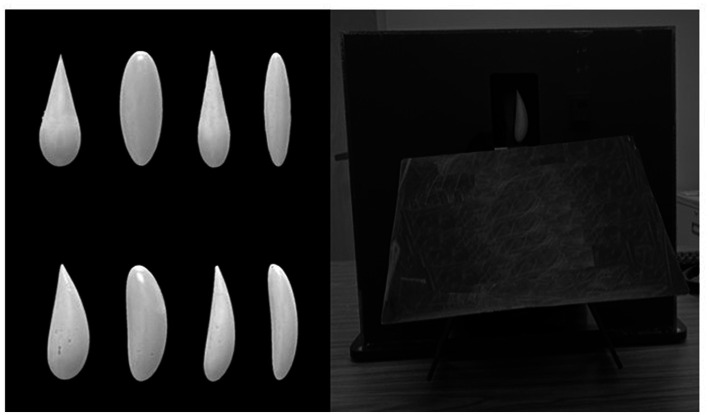
Novel objects and apparatus used in the experiment.

We used eight unique objects varying in curvature, thickness, and tapering that were psychophysically scaled by Desmarais and Dixon (2005). Objects were 81 mm long and made of light-gray polyvinyl chloride (see Figure 1).

### Apparatus

We used a 45 cm x 40 cm x 44.5 cm rotating apparatus made of black polyvinyl chloride (PVC) to present the eight three-dimensional objects. The apparatus was made of two stacked rotating cylinders, each containing evenly distributed copies of the same eight objects. In the top cylinder, the objects were obstructed from view such that the participant could only see one object in a 6.5 cm x 10 cm window. The bottom cylinder was completely obstructed from view by three sheets of black PVC, one on each side and one angled over an opening such that the participants could grasp one object with their hand. The participants were seated on one side of the apparatus, and the experimenter on the other side.

### Distractors

The verbal distractors included the 26 letters of the English alphabet in Lucida handwriting font, and the visual distractors included 26 symbols from the Yi language, an ideogram script presented in Microsoft Yi Baiti font. The distractors were presented using two separate binders and, during the experiment, the participant was shown a page containing a single distractor in 210-point size, and later a page containing a set of eight distractors – two rows of four distractors in 110-point size.

### Electronic equipment

The experiment was run on a computer using Superlab 6.0, a stimulus-delivery software (Superlab, 2020).

### Procedure

**Figure 2:**
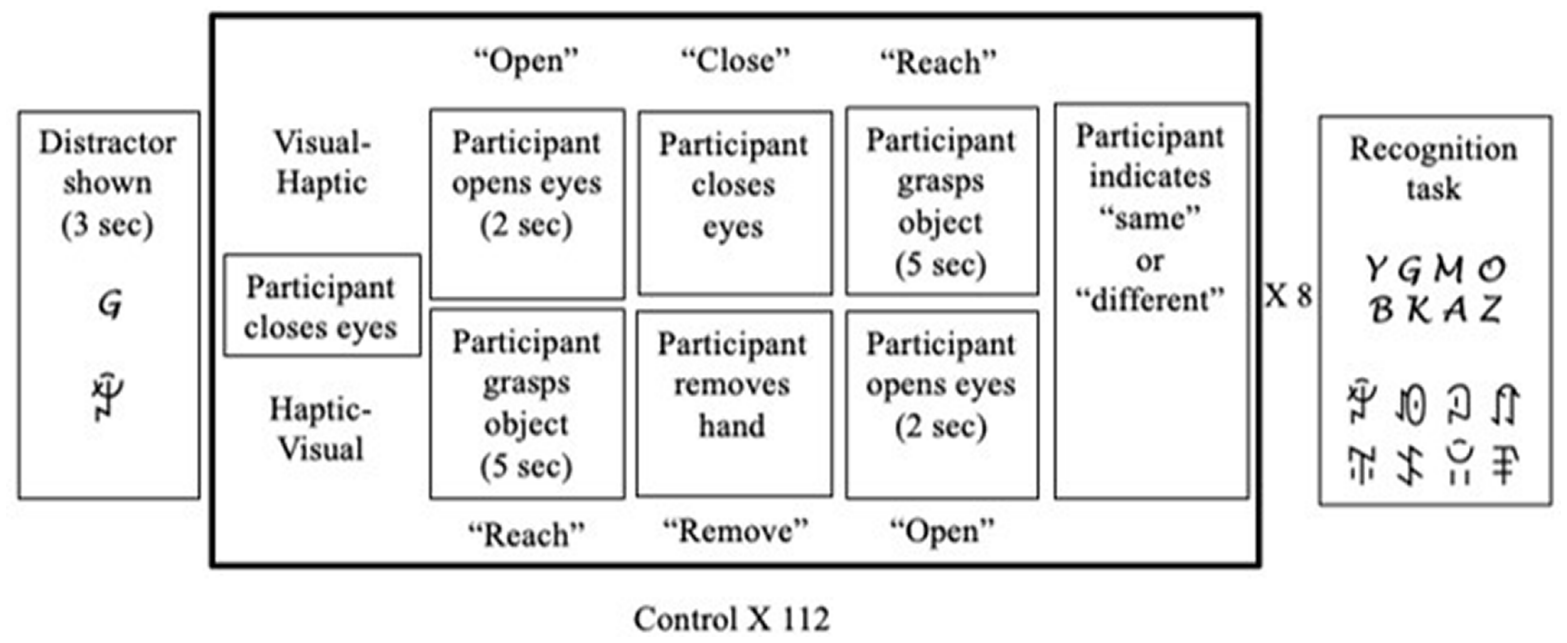
Experimental procedure.

Participants first completed the OSIVQ, followed by the matching task, which consisted of a block of visual-haptic (VH) trials and a block of haptic-visual (HV) trials. Participants were randomly assigned to either the control condition (*n* = 41), the verbal distractor condition (*n* = 42), or visual distractor condition (*n* = 44), and were randomly assigned to start with either visual-haptic or haptic visual trials. See Figure 2 for a visual description of the full experimental procedure.

### Visual-haptic trials (VH trials)

During VH trials participants viewed an object (V) and then touched an object (H). Participants were first instructed to close their eyes. Once the experimenter placed the objects for the trial, the auditory cue “open” signaled participants to open their eyes to view the object. After two seconds, the cue “close” signaled participants to close their eyes, followed by the cue “reach,” that signaled participants to reach out their hand and grasp an object placed at the bottom. Once they touched the object, participants indicated on a keyboard whether the object they touched was the same or different from the one they viewed, completing the trial and signaling the experimenter to prepare the next trial. Each of the eight novel objects were presented seven times with a second copy of the same object and seven times with a different object, for a total of 112 trials in random order.

### Haptic-visual trials (HV trials)

During HV trials participants touched an object (H) and then viewed and object (V). The HV trials were identical to the VH trials save for the order of presentation: participants touched an object for five seconds before removing their hand and viewing an object. The procedure was then repeated 112 times in a manner identical to the VH trials. For both types of trials, the recording of reaction time started at the onset of the cue for the second modality.

In the experimental conditions with interference, participants completed trials as described above with the addition of either visual or verbal distractors throughout the experiment. Before the first trial, the participants were shown a page with a single distractor for three seconds before closing their eyes to begin the matching trial. This was repeated for each odd numbered trial (1, 3, 5, 7) for a total of four distractors. After eight trials were completed, the participant was shown a page containing two rows of four characters, including the four distractors previously viewed and four other characters. They were asked to indicate which distractors were presented during the preceding trials, and the experimenter recorded the number of distractors correctly recalled. The set of eight trials followed by a distractor recognition task was repeated fourteen times to complete the block of trials. Testing time was approximately 2 h.

## Results

The data of four participants was excluded for discontinuing the study. Three participants were in the visual distraction condition (in the VH modality order), and one participant was in the verbal distraction condition (in the HV modality order), and as a result there were 41 participants per condition. Prior to analyses, the data was trimmed recursively at three standard deviations, such that 4 % of data points were removed overall.

### Distribution of OSIVQ scores

We entered OSIVQ scores in a 3 (OSIVQ subscale) X 3 (distraction type) mixed design Analysis of Variance (ANOVA) where distractor type was the between subject factor using JASP, a statistical program that performs both classic and Bayesian analyses (JASP Team, 2022). The analysis only revealed a main effect of scale, *F* (2, 240) = 51.297, *p* < 0.001, *partial η^2^* = 0.299. Paired-samples post-hoc comparisons showed that scores were significantly different across all three scales: participants scored higher on the object-visualizer subscale (*M* = 3.44) than on the verbalizer subscale [*M* = 2.95, *t* (122) = 6.663, *p* < 0.001] or the spatial-visualizer subscale [*M* = 2.67, *t* (122) = 9.485, *p* < 0.001], which was also significantly lower than their score on the verbalizer scale [*t* (122) = 3.610, *p* < 0.001]. We followed up with a Bayesian mixed design ANOVA, for which the strongest model also included only a main effect of scale type (BF_10_ = 3.264 × 10^19^) providing strong evidence for the alternative hypothesis (Wagenmakers et al., 2018). Again, paired-samples Bayesian post-hoc comparisons showed that scores were significantly different across all three scales: participants scored higher on the object-visualizer subscale (*M* = 3.44) than on the verbalizer subscale (*M* = 2.95, BF_10_ = 1.157 × 10^7^) or the spatial-visualizer subscale (*M* = 2.67, BF_10_ = 2.370 × 10^13^), which was also significantly lower than their score on the verbalizer scale, BF_10_ = 42.837. Importantly, the Bayes factor (BF) for the interaction between the two variables (BF_01_ = 40.536) provides very strong support for the null hypothesis for the interaction, indicating that subscale scores were not significantly different between individuals in the three distraction conditions.

### Distractor accuracy

We entered participants’ average distraction recognition accuracy into an independent samples t-test. Participants presented with verbal distractors recognized more distractors (*Mean recall* = 3.91/4) than participants presented with visual distractors (*Mean recall* = 3.75/4), *t* (80) = 2.512, *p* = 0.014, *Cohen’s d* = 0.229. The Bayesian t-test confirmed this and only indicated moderate support for the alternative hypothesis, BF_10_ = 3.398.

### Reaction time

**Figure 3:**
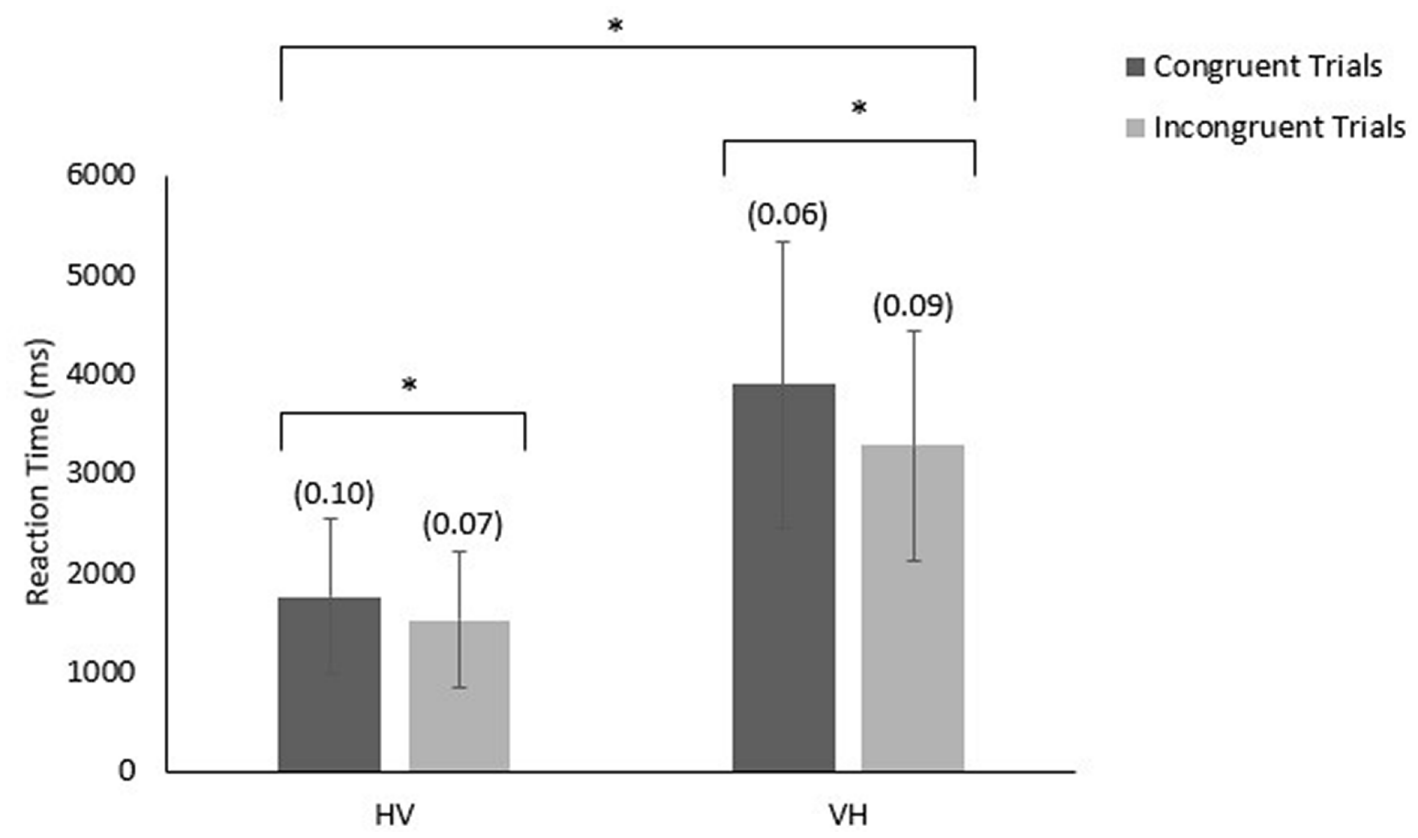
Mean reaction time (and error rates) in response to congruent and incongruent HV and VH trials.

Reaction times were measured from the onset of the cue to either look at or grasp the second object. We entered participants’ reaction time into a 2 (congruence) × 2 (modality order) × 3 (distractor type) mixed ANOVA where distractor type was the only between subject factor. The analysis revealed a main effect of congruence, *F* (1,120) = 136.427, *p* < 0.001, partial η^2^ =. 325; a main effect of modality order, *F* (1,120) = 413.287, *p* < 0.001, partial η^2^ = 0.775, and an interaction between the two variables, *F* (1,120) = 32.047, *p* < 0.001, partial η^2^ = 0.211. The complementary Bayesian ANOVA’s strongest model also included a main effect of congruence, a main effect of modality order, and an interaction between congruence and modality order (see Figure 3). The BF_10_ for this model was 1.866 × 10^104^, suggesting that these data were 1.866 × 10^104^ times more likely to occur under the model where only these effects/interactions were present, providing very strong evidence for the alternative hypothesis. Generally, participants responded faster to incongruent trials (*Mean RT* = 2,397 ms) compared to congruent trials (*Mean RT* = 2,824 ms), and they responded faster when responding to an object they saw (*Mean RT* = 1,637 ms) compared responding to an object they touched (*Mean RT* = 3,584 ms).

We analyzed the interaction using Bayesian paired samples t-tests. For VH trials, there was a difference of approximately 600 ms between congruent (*Mean RT* = 3,890 ms) and incongruent (*Mean RT* = 3,279 ms) trials (BF_10_ = 7.885 × 10^14^) suggesting that the data were 7.885 × 10^14^ times more likely to be observed under the alternative hypothesis and providing very strong evidence for the alternative hypothesis. In contrast, for HV trials, there was a difference of approximately 200 ms between congruent (*Mean RT* = 1758 ms) and incongruent (*Mean RT* = 1,516 ms) trials (BF_10_ = 7.749 × 10^7^) suggesting that the data were 7.749 × 10^7^ times more likely to be observed under the alternative hypothesis and providing very strong evidence for the alternative hypothesis. Therefore, participants generally demonstrated a smaller difference in reaction time between congruent and incongruent trials for trials where they were responding to an object they saw.

### Errors

We entered participants’ proportion of errors into a 2 (congruence) X 2 (modality order) X 3 (distractor type) mixed ANOVA where the distractor type was the only between subject factor. The analysis revealed a main effect of modality order *F* (1,120) = 4.326, *p* < 0.05, partial η^2^ = 0.035, and an interaction between the congruence and modality order, *F* (1,120) = 39.826, *p* < 0.001, partial η^2^ = 0.249. The complementary Bayesian ANOVA’s strongest model included a main effect of congruence, a main effect of modality order, and an interaction between congruence and modality order. The BF_10_ for this model was 2.347 × 10^4^, suggesting that the data were 2.347 × 10^4^ times more likely to be observed under the alternative hypothesis and providing very strong evidence for the alternative hypothesis. We analyzed the interaction between congruence and order of modalities using Bayesian paired samples t-tests (see Figure 3). For VH trials, participants made more matching errors for incongruent trials (*Mean proportion of errors* = 0.09) compared to congruent trials (*Mean proportion of errors* = 0.06) (BF_10_ = 3538.363, providing very strong evidence for the alternative hypothesis). For HV trials, participants made more matching errors for congruent trials (*Mean proportion of errors* = 0.10) compared to incongruent trials (*Mean proportion of errors* = 0.07); the BF_10_ was 7.686, providing only moderate evidence for the alternative hypothesis. Furthermore, when responding to congruent trials participants made fewer errors for VH trials (*Mean proportion of errors = 0.06*) than for HV trials (*Mean proportion of errors = 0.10*) (BF_10_ = 6817.178), providing very strong evidence for the alternative hypothesis); while when responding to incongruent trials, participants made more errors for VH trials (*Mean proportion of errors = 0.09*) than for HV trials (*Mean proportion of errors = 0.07*) (BF_10_ = 1220.952), providing very strong evidence for the alternative hypothesis.

### Linear regression

To determine whether cognitive styles predicted reaction time, we conducted four linear regression analyses, one for each of the experimental conditions (HV congruent, HV incongruent, VH congruent, and VH incongruent) for which OSIVQ subscale scores were used as predictors. The model was significant for HV congruent trials *F* (4, 118) = 2.68, *p* = 0.035 and accounted for 8.3% of the variability in reaction time. The model included two significant predictors: scores on the verbalizer scale (*b* = −325.49, *SE* = 133.61, *p* = 0.016) and scores on the spatial visualizer scale (*b* = −244.51, *SE* = 106.72, *p* = 0.024). Likewise, the model was significant for VH congruent trials *F* (4, 118) = 2.74, *p* = 0.032 and accounted for 8.5% of the variability in reaction time. Only scores on the verbalizer scale were significant predictors of performance (*b* = −772.03, *SE* = 250.54, *p* = 0.003). The model was not significant for HV incongruent trials *F* (4, 118) = 1.96, *p* = 0.106 and accounted for 6.2% of the variability in reaction time. However, scores on the verbalizer scale were significant predictors of performance (*b* = −297.67, *SE* = 119.53, *p* = 0.014). Similarly, the model was only marginally significant for VH incongruent trials *F* (4, 118) = 2.22, *p* = 0.071 and accounted for 7% of the variability in reaction time. Again, scores on the verbalizer scale significantly predicted performance (*b* = −549.41, *SE* = 201.43, *p* = 0.007). Overall, higher scores on the verbalizer subscale were associated with faster reaction times across all trial types and distraction conditions.

The same analyses were carried out for proportion of errors; no significant regression models were observed.

## Discussion

We investigated whether participants’ cognitive styles predicted performance in a visuo-haptic cross modal object matching task. Participants completed the task without interference, or with verbal or visual interference. Though we did not observe any interference from the distractors, our analysis provides evidence for a verbal code. The verbalizer subscale predicted reaction time on the matching task in all experimental conditions: higher scores were associated with faster reaction times. Overall, this supports the idea that object processing is facilitated by a verbal code, since the verbalizer scale indicates an increased ability to process verbal information, and this predicted better performance on the matching task. In other words, it seems that participants are using verbal representations, and therefore verbal processing ability then contributed to object processing.

The notion that object processing involves a verbal code is consistent with research findings for long-term memory representations (Lacey and Campbell, 2006; Desmarais et al., 2017a; Desmarais and Penrose, 2021) and short-term memory representations (Postle et al., 2005; Santana and Galera, 2014). For example, Desmarais et al. (2017a) instructed participants to learn to produce the actions associated with novel objects that were identified by a nonword label while a different group learned the same object-action associations without a label identifier. Participants made more action production errors based on object similarity in the condition with verbal labels, suggesting that a verbal component might facilitate binding information in long-term memory. Evidence for the use of a verbal code in short-term memory can be found in the work of Santana and Galera (2014), who observed that the verbal dimension of a letter arrangement was bound to the information participants intended to encode even when they were instructed to ignore it, which suggests that working memory may recruit verbal representations as well.

Studies using distractors also suggest that objects are encoded using a verbal code (Postle et al., 2005; Lacey and Campbell, 2006; Desmarais and Penrose, 2021), as researchers observed that verbal distractors interfered with object processing. However, in our study neither distractors interfered with participants’ matching performance. It is possible that the matching task engaged participants’ working memory while the distractors were being stored in long-term memory, minimizing the impact of those distractors on performance. In the matching task, there were only a few seconds before the two objects had to be compared, while the distractors had to be held in memory over approximately three minutes (over eight matching trials). Though we expected letter rehearsal to interfere with matching, the transfer into long-term memory could explain the lack of impact.

Though Lacey and Campbell (2006) used a matching task similar to ours, they used an audio recording as a verbal distractor presented simultaneously with encoding. Though participants did not need to attend to the distractors, processing this simultaneous verbal information would interfere with the object processing in short-term memory, and perhaps serve as competition for the object information to be encoded in long-term memory for identification. This is similar to why Postle et al. (2005) observed interference from verbal distractors in an n-back task. Notably, Postle et al. (2005) presented their verbal distractors between stimuli: participants were presented with a shape, performed the distractor task, and then another shape which they had recognize as being identical to the previous shape or not. Since the distractors were presented and identified while the participant would be rehearsing the representation of the first shape, the distractors would have served as competition for the stimuli in short-term memory. Largely, it seems that the differences between the tasks in the current study and the tasks in previous studies would result in the observed differences in verbal interference. In contrast, Desmarais and Penrose (2021) observed interference from verbal distractors in an object recognition task: participants learned to recognize objects by sight or touch while being presented with verbal interference or visual interference. The identification task was more like a long-term memory task since participants encoded the objects during a set of learning trials, and then at a later point in time (greater than 30 s) were asked to identify the objects by name. Though the distractor task was similar to the one used in the current study, both the object representations and the distractors would have been competing to enter long-term memory. In this case, it makes sense that interference was found from the verbal distractors – especially since the objects were being identified by name, another piece of verbal information.

### Limitations and future directions

We tested participants using simple novel objects that differed from one another on curvature, tapering, and thickness. These simple objects may be more easily formed into verbal or visual representations, and these results may not generalize to complex objects that possess more intricate differences. Similarly, familiar objects for which representations already exist in memory and that are identified by single label might rely even more strongly on a verbal code. Future research should investigate memory representations for complex and familiar objects.

### Summary and conclusion

In conclusion, participants’ scores on the verbalizer scale consistently predicted performance, suggesting that cross-modal object processing in short-term memory may be facilitated by a verbal code. However, our results may be stimulus-specific, and different patterns of performance might be observed with more complex or familiar objects.

## Data availability statement

The raw data supporting the conclusions of this article will be made available by the authors, without undue reservation.

## Ethics statement

The studies involving humans were approved by Mount Allison University Research Ethics Board. The studies were conducted in accordance with the local legislation and institutional requirements. The participants provided their written informed consent to participate in this study. Written informed consent for participation was not required from the participants’ legal guardians/next of kin because undergraduate students are sometimes 17 but allowed to participate as adults.

## Author contributions

GD contributed to the conception and design of the study. YIS organized the database. GD and KE performed the statistical analysis. KE wrote the first draft of the manuscript. GD, KE, and MS wrote sections of the manuscript. All authors contributed to the article and approved the submitted version.
